# Phylogenetic Analysis of a Swine Influenza A(H3N2) Virus Isolated in Korea in 2012

**DOI:** 10.1371/journal.pone.0088782

**Published:** 2014-02-11

**Authors:** Jin Il Kim, Ilseob Lee, Sehee Park, Sangmoo Lee, Min-Woong Hwang, Joon-Yong Bae, Jun Heo, Donghwan Kim, Seok-Il Jang, Kabsu Kim, Man-Seong Park

**Affiliations:** 1 Department of Microbiology, College of Medicine, Hallym University, Chuncheon, Gangwon-do, Republic of Korea; 2 Center for Medical Science Research, College of Medicine, Hallym University, Chuncheon, Gangwon-do, Republic of Korea; 3 School of Equine Science, Cheju Halla University, Jeju, Jeju-do, Republic of Korea; Centers for Disease Control and Prevention, United States of America

## Abstract

Influenza A virus (IAV) can infect avian and mammalian species, including humans. The genome nature of IAVs may contribute to viral adaptation in different animal hosts, resulting in gene reassortment and the reproduction of variants with optimal fitness. As seen again in the 2009 swine-origin influenza A H1N1 pandemic, pigs are known to be susceptible to swine, avian, and human IAVs and can serve as a ‘mixing vessel’ for the generation of novel IAV variants. To this end, the emergence of swine influenza viruses must be kept under close surveillance. Herein, we report the isolation and phylogenetic study of a swine IAV, A/swine/Korea/PL01/2012 (swPL01, H3N2 subtype). After screening nasopharyngeal samples from pigs in the Gyeongsangnam-do region of Korea from December 2011 to May 2012, we isolated the swPL01 virus and sequenced its all of 8 genome segments (polymerase basic 2, PB2; polymerase basic 1, PB1; polymerase acidic, PA; hemagglutinin, HA; nucleocapsid protein, NP; neuraminidase, NA; matrix protein, M; and nonstructural protein, NS). The phylogenetic study, analyzed with reference strains registered in the National Center for Biotechnology Information (NCBI) database, indicated that the swPL01 virus was similar to the North American triple-reassortant swine strains and that the HA gene of the swPL01 virus was categorized into swine H3 cluster IV. The swPL01 virus had the M gene of the triple-reassortant swine H3N2 viruses, whereas that of other contemporary strains in Korea was transferred from the 2009 pandemic H1N1 virus. These data suggest the possibility that various swine H3N2 viruses may co-circulate in Korea, which underlines the importance of a sustained surveillance system against swine IAVs.

## Introduction

Influenza A viruses (IAVs) are respiratory pathogens of a genus of the family *Orthomyxoviridae* and infect many animal species [1]. The wide range of IAV hosts, which expanded from the natural reservoir in aquatic birds through domestic poultry, pigs, humans, cats, marine mammals, and bats, can affect the evolution of IAVs. By antigenic drift, new IAV variants evade the immune barrier of existing hosts [2], [3]. Sometimes, IAVs go through antigenic shift by genetic reassortment among evolutionary lineages [1], [4]. These all expand the host range of IAVs and change the evolutionary dynamics of IAVs in different species.

Pigs are known as an intermediate host of IAVs [5]. Because NeuAc-α2,3-Gal- and NeuAc-α2,6-Gal-linked sialic acids are present in swine respiratory tracts, both human and avian IAVs can readily infect pigs [6]. When these viruses co-infect a single cell, progeny virions may have more opportunities for their gene selection, and consequently, pigs can serve as a wonderful melting pot for genetic reassortment and adaptation of IAVs [7]. Pigs are also an important host for the evolution of IAVs. Since 1918 influenza A H1N1 pandemic in humans, antigenically similar viruses have also transferred into swine [8]. The so-called classical swine (CS) H1N1 virus is still circulating and causing respiratory diseases in pigs. In 1998, the distinct swine H3N2 virus caused epizootic in North American pigs [9], [10]. This virus retained gene constellation of avian (PB2 and PA), human (PB1, HA, and NA), and CS lineages (NP, M, and NS) [11]. Since then, the triple-reassortant virus has gone through multiple reassortment events and thus it diverged into various evolution routes of IAVs [12]. During these reassortment events, the triple-reassortant internal genes (TRIG) have been a gene cassette for accepting different HA and/or NA genes from other contemporary human and swine IAVs [13]. Until today, these swine IAVs continue to circulate and to establish their individual evolution not only in pigs but also in other susceptible hosts [14].

Recent human infections with swine H3N2 variants (H3N2v) have been another result of the IAV reassortment that occurred in pigs. By accepting the M gene segment from the 2009 pandemic influenza A H1N1 (pH1N1) virus, the H3N2v virus was generated on the backbone of the TRIG H3N2 virus [15]. The subsequent phylogenetic study suggested that more than four reassortment events between the TRIG H3N2 and pH1N1 viruses have occurred since the pH1N1 outbreak in 2009 [16]. Generally affecting children, the novel H3N2v virus resulted in limited, but possible, human-to-human transmission [17] and a large number of previously unnoticed human cases [18]. These issues confirm the importance of a close comprehensive global surveillance system to monitor the emergence of swine-origin IAV variants [19].

In this study, we report the phylogenetic relations of swine H3N2 influenza virus isolated from pigs in Korea between December 2011 and May 2012. Phylogenetic analysis indicated that the virus was similar to the North American TRIG H3N2 strains without a reassortment trace of the M segment from the pH1N1 virus. The HA gene of this virus was phylogenetically grouped into swine H3 cluster IV.

## Materials and Methods

### Ethics statement

This study was conducted in strict accordance with the recommendations in the Guide for the Care and Use of Laboratory Animals of the Animal, Plant, and Fisheries Quarantine and Inspection Agency of Korea. Based on agreement between the Office of University-Industry Cooperation of Hallym University and the Gyeongsangnam-do Livestock Promotion Institute, the experimental protocol was approved by the Institutional Animal Care and Use Committee (IACUC) of Hallym University (permit number: Hallym 2012-95). To minimize unnecessary animal suffering, the veterinary inspector collected nasopharyngeal samples only from the pigs exhibiting respiratory symptoms, and the samples were used for the virus isolation and phylogenetic characterization.

### Sampling and isolation of virus

Based on the Hallym University IACUC permission, Hallym 2012-95, 108 nasopharyngeal samples were collected from pigs between December 2011 and May 2012 in Gyeongsangnam-do, a southeastern province of Korea. Carried in universal viral transport media (BD, Franklin Lakes, NJ), samples were prepared for the virus isolation. The prepared samples were inoculated into the Madin-Darby canine kidney (MDCK) cell monolayer for 1 h at 37°C. The cells were washed and maintained with growth media for 48–72 h. Viral growth was determined by the morphological changes (cytopathic effects) in the MDCK cells and by a hemagglutination assay of cell supernatants using 0.5% turkey red blood cells (Rockland Immunochemicals Inc.; Gilbertville, PA). The double-positive sample was purified by a standard plaque assay in the MDCK cells and prepared after propagation in fertile chicken eggs.

### Genomic sequence analysis

To determine the genomic sequences of the isolated virus, the viral RNAs of egg-grown samples were extracted using a QIAamp Viral RNA Mini Kit (Qiagen, Venlo, Netherlands). After reverse transcriptase PCR (RT-PCR), the DNA products were used for the sequence readouts. More than three clones per genomic segment were used for the sequence analysis. This virus was subtyped as a H3N2 strain and named A/swine/Korea/PL01/2012 (swPL01). Eight genomic sequences of the swPL01 virus were registered in the NCBI database, with the accession numbers KF382726 to KF382733 from the PB2 gene to the NS.

### Phylogenetic and genetic distance analysis

For the phylogenetic study, the NCBI-registered full-length nucleotide sequences of swine H3N2 viruses were used as references (the total number of full-length sequences after collapsing the identical sequences as of release date June 29, 2013 is as follows: PB2, 341; PB1, 358; PA, 354; HA, 556; NP, 352; NA, 550; M, 400; and NS, 331). By adding various lineage defining sequences or removing redundant sequences, we analyzed the phylogenetic characteristics of the swPL01 virus. The nucleotide sequences of lineage defining human H1N1 (huH1N1; A/New York/494/2002), huH3N2 (A/New York/101/2002), pH1N1 (A/California/04/2009, A/Korea/01/2009, A/Netherlands/602/2009, A/swine/Hong Kong/2995/2009, and A/swine/Hong Kong/189/2010) [20], CS H1N1 (A/swine/Hong Kong/5683/1999, A/swine/Hong Kong/NS143/2000, A/swine/Hong Kong/8690/2001, and A/swine/Hong Kong/227/2002) [20], Eurasian avian-like (EA) H1N1 (A/swine/Hong Kong/638/2003, A/swine/Hong Kong/NS129/2003, A/swine/Hong Kong/435/2007, A/swine/Hong Kong/245/2009) [20], TRIG H1N2 (A/swine/Ontario/52156/2003, A/swine/Ontario/48235/2004, A/swine/Oklahoma/010226-16/2008, A/swine/Texas/008648/2008, A/swine/Hong Kong/1435/2009) [20], [21], H3N2v (A/Indiana/10/2011 and A/Maine/06/2011) [16], and avian H1N1 (avH1N1; A/mallard/Maryland/02-181/2002) and avH3N2 (A/blue-winged teal/Ohio/908/2002) viruses were included in the analysis, except for the HA and NA sequences. For HA gene analysis, only the nucleotide sequences of swine H3N2 and H3N2v viruses were included. For NA gene analysis, only the nucleotide sequences of swine H3N2, TRIG H1N2, and H3N2v viruses were included. Using MEGA5 [22], each sequence of the swPL01 virus was aligned with that of the references using MUSCLE by the unweighted pair group method with the arithmetic mean [23]. The phylogenetic tree was inferred using the Maximum Likelihood (ML) method by obtaining the initial tree(s) for the heuristic search based on the neighbor-joining method through a matrix of pairwise distances evaluated by the maximum composite likelihood (MCL) approach. The bootstrap scores were determined from 500 replicates and are shown below the branches [24]. The sequences that met our bootstrap score standards (>95% for PB2, PB1, PA, and NP or >60% for HA, NA, M, and NS among the sequences registered since the late 1990s) and that were located in close proximity were recalculated with the Korean swine H3N2 and lineage defining sequences for the final ML tree evaluation. Some HA sequences published previously in 2008 [25] and 2013 [26] were included in the final HA tree for the differentiation of HA clusters. The horizontal distances are the nucleotide differences, which were proportional for the number of changes per site. The genetic distances were determined with MEGA5 by the MCL method using bootstrap estimation (500 replicates).

## Results

### Phylogenetic analysis of the polymerase genes

**Figure 1:**
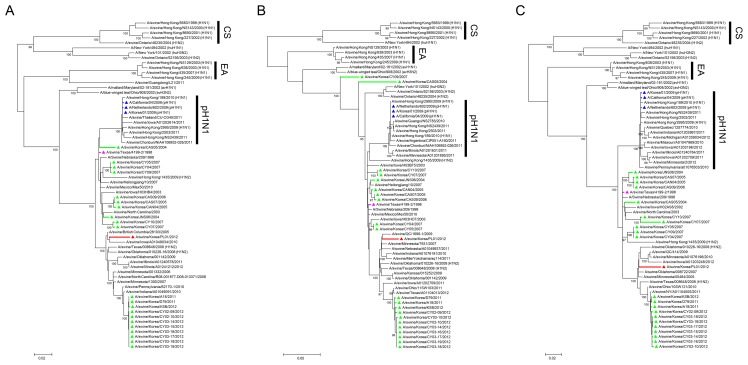
The phylogenetic relationships of polymerase segments. The evolutionary phases of the PB2 (A), PB1 (B), and PA (C) segments of the swPL01 virus were inferred with those of NCBI-registered reference sequences by the ML method using MEGA5 [22]. The sequences with more than 95% bootstrap scores among the evolution branches of the swTx/98 virus and those of reference were used for the final ML tree evaluation. Virus subtypes were indicated inside parenthesis along with the respective abbreviation of hosts. Lineage definitions were indicated as: CS, classical swine; EA, Eurasian avian-like; and pH1N1, 2009 pandemic influenza A H1N1. The colors represent the following viruses: pink, A/swine/Texas/4199-2/1998 (swTx/98); green, Korean swine H3N2; red, A/swine/Korea/PL01/2012 (swPL01); and blue, 2009 pandemic influenza H1N1 viruses.

We first analyzed the phylogenetic relations of three polymerase gene segments of the swPL01 virus with other NCBI-registered sequences. In the ML trees, the polymerase genes of Korean swine H3N2 viruses appeared to have evolved from those of the swTx/98 virus (Figure 1). Although some divergences were found in the PB1 gene (A/swine/Korea/CAS05/2004 and A/swine/Korea/CY09/2007) (Figures 1B and S2), the polymerase genes of the Korean isolates were clustered in chronological order. A number of the selected TRIG H3N2 strains identified in Southeast Asia, South America, and North America appeared to reassort with pH1N1 viruses and consisted of different sub-lineages from the swTx/98 virus (Figure 1). Especially for the PA gene, the phylogenetic analysis indicated that nine North American TRIG H3N2 viruses were involved in reassortment events with pH1N1 strains (Figures 1C and S3).

All of the polymerase genes of the swPL01 virus appeared to be evolved from the genes of the swTx/98 virus. However, the swPL01 virus was categorized into a different clade from the other contemporary Korean isolates (Figure 1). These relations were easily appreciated in the ML trees drawn with the overall reference sequences and also seen in maximum parsimony (MP) and neighbor joining (NJ) trees of the selected sequences (Figures S1, S2, and S3). In sequence analysis, the PB2 of A/swine/Ontario/33853/2005 showed the greatest sequence similarity (97.5%) with that of the swPL01 virus whereas maximum 95.7% similarity was seen with the Korean isolates (Table 1). For the PB1 and PA genes, North American isolates in 2005 shared maximum similarity (PB1, 97.8% with A/swine/Minnesota/00611/2005; 97.4% with A/swine/Ontario/33853/2005) (Table 1). On the other hand, 95.7% and 95.9% were the highest sequence similarities found with the PB1 and PA genes of the Korean isolates, respectively (Table 1). Considered together, these findings suggest that the swPL01 virus retains polymerase genes that drifted from the swTx/98 virus but it is phylogenetically different from those of other contemporary Korean strains.

**Table 1:** Sequence similarity and maximum identity of the swPL01 virus with the Korean and other swine H3N2 viruses.

	% pairwise sequence similarity of the swPL01 virus to each gene of the Korean isolates							
Virus	PB2	PB1	PA	HA	NP	NA	M	NS
A/swine/Texas/4199-2/1998	**96.0**	**96.0**	95.6	90.9	**96.3**	93.5	96.6	96.5
A/swine/Korea/JNS06/2004	95.0	95.5	94.7	93.1	95.3	92.1	81.5	U/A
A/swine/Korea/CAS05/2004	94.4	91.4	94.4	91.2	95.6	93.2	95.9	96.1
A/swine/Korea/CAN04/2005	94.6	95.5	94.6	89.9	95.7	93.0	**97.5**	**97.0**
A/swine/Korea/CAS07/2005	94.6	95.7	94.6	89.9	95.6	92.6	97.4	**97.0**
A/swine/Korea/CAS09/2006	94.7	95.7	94.7	89.9	95.7	92.7	97.4	96.9
A/swine/Korea/CY04/2007	94.9	95.5	94.6	91.1	**96.3**	93.0	96.9	93.1
A/swine/Korea/CY05/2007	94.9	95.5	94.6	91.1	**96.3**	92.8	96.9	93.1
A/swine/Korea/CY07/2007	94.5	94.7	92.2	91.3	95.0	92.2	95.6	95.0
A/swine/Korea/CY09/2007	94.9	92.1	94.5	91.1	96.2	93.1	96.9	93.1
A/swine/Korea/CY10/2007	94.5	94.8	93.8	92.3	95.0	92.2	95.7	95.1
A/swine/Korea/A18/2011	95.7	95.5	**95.9**	94.7	94.6	**94.0**	87.0	95.8
A/swine/Korea/D79/2011	95.7	95.6	**95.9**	**94.8**	94.6	**94.0**	87.1	95.7
A/swine/Korea/CY02-09/2012	95.7	95.5	95.7	94.6	95.8	93.8	87.4	95.7
A/swine/Korea/CY02-10/2012	95.7	95.6	95.8	94.4	95.4	93.8	87.3	95.6
A/swine/Korea/CY03-14/2012	95.7	95.6	**95.9**	94.4	95.5	93.8	87.3	95.8
A/swine/Korea/CY03-15/2012	95.7	95.6	**95.9**	94.4	95.6	93.4	87.3	95.8
A/swine/Korea/CY03-16/2012	95.7	95.6	**95.9**	94.4	95.6	93.8	87.3	95.8
A/swine/Korea/CY03-17/2012	95.7	95.6	**95.9**	94.4	95.6	93.8	87.3	95.8
A/swine/Korea/CY03-18/2012	95.7	95.6	95.8	94.3	95.5	93.8	87.3	95.8
A/swine/Korea/CY03-19/2012	95.7	95.6	**95.9**	94.4	95.7	93.8	87.3	95.8
A/swine/Korea/KSB/2012	95.7	95.6	**95.9**	94.5	94.5	93.5	87.3	95.7
CA04	93.8	93.8	92.6	53.0	92.9	51.0	87.3	93.9
K09	93.9	93.8	92.7	53.1	92.8	50.9	87.3	93.8
NL602	93.8	93.8	92.7	53.1	94.1	51.0	87.3	93.9
Virus of max. identity (%)	A/swine/Ontario/33853/2005	A/swine/Minnesota/00611/2005	A/swine/Ontario/33853/2005	A/swine/Ohio/11SW347/2011	A/swine/Minnesota/00611/2005	A/swine/Quebec/4001/2005	A/swine/Alberta/14722/2005	A/swine/Manitoba/12707/2005
					A/swine/Ontario/33853/2005		A/swine/British Columbia/28103/2005	A/swine/Ontario/3385/2005
							A/swine/Manitoba/12707/2005	A/swine/Quebec/4001/2005
							A/swine/Manitoba/12707/2005	
							A/swine/Minnesota/00611/2005	
							A/swine/Ontario/33853/2005	
							A/swine/Quebec/4001/2005	
	97.5	97.8	97.4	96.6	98.1	96.9	99.1	97.9

*Bold numbers indicate the maximum sequence similarity to the Korean isolates.

### Phylogenetic analysis of the HA and NA genes

**Figure 2:**
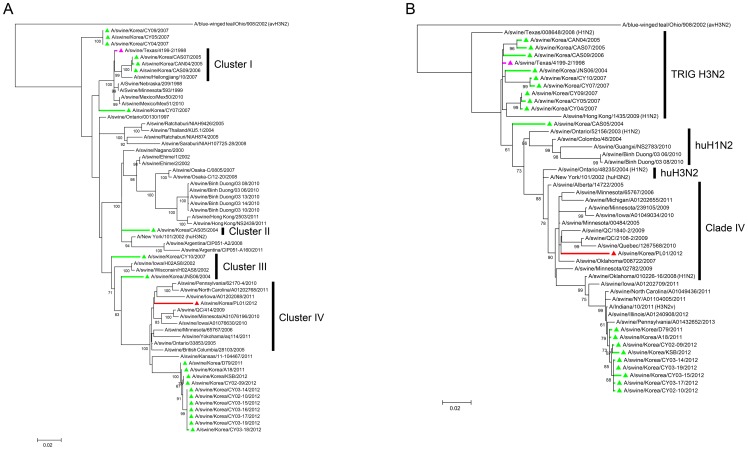
The phylogenetic relationships of surface glycoprotein segments. The evolutionary phases of the HA (A) and NA (B) segments of the swPL01 virus were inferred using the method described above (see the legend in Figure 1). The sequences with more than 60% bootstrap scores among the evolution branches of the swTx/98 virus and those of reference were used for the final ML tree evaluation. The HA phylogenetic groups were indicated as cluster I, II, III, and IV. The NAs were indicated as TRIG H3N2, human H1N2 (huH1N2), human H3N2 (huH3N2), and clade IV. The colors represent the following viruses: pink, A/swine/Texas/4199-2/1998 (swTx/98); green, Korean swine H3N2; and red, A/swine/Korea/PL01/2012 (swPL01).

We then analyzed the phylogenetic characteristics of the swPL01 HA and NA genes. In the ML tree of the overall sequences, the HA of swPL01 (indicated as c in Figure S4) virus was distant from those of other contemporary Korean viruses (b, d in Figure S4). We confirmed this phylogenetic distance with the selected HA sequences. In the ML (Figure 2A), MP and NJ (Figure S4) trees, each cluster (clusters I, II, III, and IV) of swine H3 HAs diverged from that of the swTx/98 virus [25]–[27]. In these group denotations, three Korean HAs between 2005–2006 were classified into cluster I, with the parental swTx/98 HA. The HAs of the 2007 Korean isolates consisted of subsequent tertiary and quaternary branches from the HA of the swTx/98 virus whereas that of the A/swine/Korea/CY10/2007 virus that was classified into cluster III in the MP and NJ tree (Figure S4). Farther down in the tree, two Korean HAs in 2004 were categorized as clusters II (A/swine/Korea/CAS05/2004) and III (A/swine/Korea/JNS06/2004), respectively. Under the trunk of cluster III, various HAs were diverged into two large clades. These constituted cluster IV [28], and the HA of the swPL01 virus was included in the upper clade group with others from North America whereas Korean HAs isolated from 2011–2012 were in the lower clade (Figures 2A and S4). In a similar manner to that of the phylogenetic relations of polymerase genes unveiled in Figure 1, the swPL01 HA was located in one of the branches of the cluster IV phylogenetic trunk shared with the other contemporary Korean HAs, but it was categorized in a different group (Figures 2A and S4). These differences were also apparent in the ML tree using the full-length HA sequences of Asian avian, human, and swine H3N2 viruses and North American avian and swine H3N2 viruses (Figure S9). Similarly above, the HA of the swPL01 virus was included in the completely different clade from those of other Korean isolates in the same swine H3 HA trunk. Amino acid differences also explained these phylogenetic distances (Table S1). Compared with the HAs of other Korean isolates, we found 35 amino acid mutations (35/567, 6.17%) from that of the swPL01 virus. 27 mutations (27/345, 7.83%) were located in the HA1 region, and 8 (8/222, 3.6%) were in the HA 2. By concurrent E292N and N294S mutations, the HA of swPL01 virus gained one more potential N-linked glycosylation (NLG; N-Xaa-S/T, any amino acid for Xaa except proline) at HA residue 292 (Table S1). The HA of the A/swine/Ohio/11sw347/2011 virus exhibited the highest sequence similarity (96.6%) with that of the swPL01 virus (Table 1). On the other hand, maximum 94.8% similarity was found with the HAs of the Korean swine H3N2 viruses (Table 1).

The NAs of TRIG H3N2 viruses diverged into various clades, which resulted from reassortments with seasonal human viruses (Figures S6 and S9) [21]. The reassortment event in 1998 introduced human HA, NA, and PB1 genes into the swine influenza viruses and was attributed to the establishment of the TRIG H3N2 lineage in pigs [9]. The later reassortment of TRIG H3N2 viruses occurred with the NA of human H3N2 viruses around 2001–2002. This second reassortment established another NA phylogenetic lineage, clade IV, into swine NA evolution [21]. In our phylogenetic tree of the NA genes, a similar pattern was observed (Figures 2B and S6). Evolved from the NA of the swTx/98 virus, various branch lines were inferred in the N2 lineage. Some NAs were included in the clade of A/swine/Ontario/52156/2003, which could be classified into the N2 lineage of human H1N2 viruses. All of the Korean isolates from 2004–2007 were rooted in the early N2 lineage of the swTx/98 virus (Figures 2B and S6). The NAs of Korean swine H3N2 viruses in 2011–2012 were placed at the end of the evolution tree of cluster IV, right below from the human H3N2 lineage (A/swine/Ontario/48235/2004 and A/swine/Alberta/14722/2005) [21], [28]. However, the NA of the swPL01 virus was classified into a different group from that of the other contemporary Korean viruses (Figures 2B and S6) and exhibited the closest genetic similarity to that of the A/swine/Quebec/4001/2005 virus (96.9%) rather than to any other Korean isolates (less than 94.0%) (Table 1). In addition, 29 (29/470, 6.17%) amino acid differences in the NAs between the swPL01 and other Korean isolates also supported their phylogenetic distances (Table S2). These results suggest that the HA and NA genes of the swPL01 virus are phylogenetically distant from those of the other contemporary Korean isolates.

### Phylogenetic analysis of the NP, M, and NS genes

**Figure 3:**
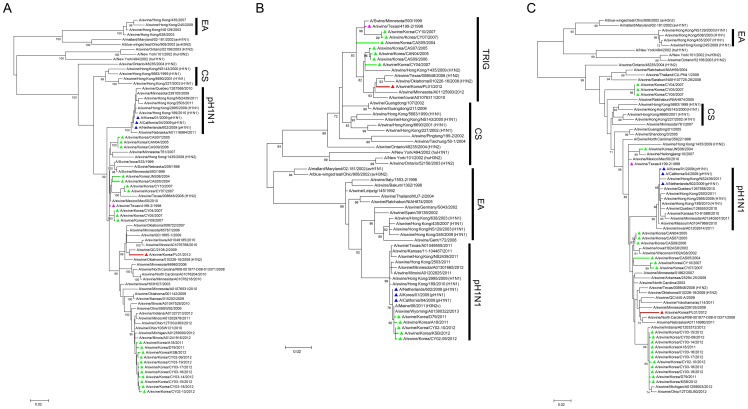
The phylogenetic relationships of NP, M, and NS segments. The evolutionary phases of the NP (A), M (B), and NS (C) segments of the swPL01 virus were inferred using the method described above (see the legend in Figure 1). The sequences with more than 95% for NP and 60% for M and NS bootstrap scores among the evolution branches of the swTx/98 virus and those of reference were used for the final ML tree evaluation. Lineage definitions were indicated as: TRIG, triple-reassortant; CS, classical swine; EA, Eurasian avian-like; and pH1N1, 2009 pandemic influenza A H1N1. The colors represent the following viruses: pink, A/swine/Texas/4199-2/1998 (swTx/98); green, Korean swine H3N2; red, A/swine/Korea/PL01/2012 (swPL01); and blue, 2009 pandemic influenza H1N1 viruses.

The phylogenetic analysis of polymerase and surface glycoprotein genes indicated the unique evolutionary position of the swPL01 virus compared with other contemporary Korean isolates (Figures 1 and 2). The overall ML trees of the NP, M, and NS genes presented a similar conclusion (Figures S5, S7, and S8). In the ML, MP, and NJ trees of the selected NP and NS sequences, the swPL01 virus appeared to be evolved from the respective swTx/98 genes but was neither classified within the same clades with other contemporary Korean isolates nor traced by interaction clues with the pH1N1 viruses (NP in Figures 3A and S5; NS in Figures 3C and S8). The NP and NS genes of the swPL01 virus had maximum 96.3% and 97.0% genetic similarities to those of Korean isolates, respectively, whereas 98.1% and 97.9% genetic similarities were observed respectively with the NP of A/swine/Minnesota/00611/2005 and A/swine/Ontario/33853/2005 viruses and the NS of A/swine/Manitoba/12707/2005, A/swine/Ontario/33853/2005/, and A/swine/Quebec/4001/2005 viruses (Table 1).

The ML tree of the swPL01 M gene suggested a different evolution pathway of TRIG H3N2 viruses in Korea (Figures S7 and S9). Most Korean TRIG H3N2 viruses in 2011-2012 appeared to retain the M gene of the pH1N1 lineage, which was derived from the EA lineage (Figures 3B and S7) [2] and termed as H3N2v [29]. However, the swPL01 virus retained the M gene signature of TRIG H3N2 viruses. Located in the phylogenetic tree of TRIG viruses, the swPL01 M segment appeared to be one of the evolutionary branch lines of the swTx/98. Some Korean swine H3N2 viruses around 2004–2007 also retained the M gene of the TRIG lineage (Figures 3B and S7). The M genes of six North American TRIG H3N2 viruses in 2005 exhibited 99.1% sequence similarity with that of the swPL01 virus; however, those of Korean swine H3N2 viruses around 2011–2012 exhibited only ∼87.3% similarity, suggesting a different evolution lineage from the swPL01 M segment (Table 1).

## Discussion

Our results demonstrate the genetic diversity of the swine H3N2 viruses circulating in Korea and the necessity of epidemiological preparedness through comprehensive surveillance. Since 2002, six different subtypes of swine IAVs (H1N1, H1N2, H3N1, H3N2, H5N2, and H9N2) have been reported in Korea (based on the NCBI-registered sequence information as of July 28, 2013) [30]–[32]. Recently, H3N2v viruses were also identified in pigs on Korean farms [33], [34]. When considering that various avian, swine, and human IAVs can infect pigs [14], swine-origin novel IAV variants in Korea may pose serious threats to local and global communities.

To maintain epidemiologic preparedness against swine IAVs, we routinely investigated nasopharyngeal samples collected from pigs in Gyeongsangnam-do, a southeastern province of Korea and isolated the swPL01 virus. In the phylogenetic analysis, this virus appeared to be one of the North American TRIG H3N2 strains. Since 1998, the TRIG cassette has provided a stable backbone for various insertion sets of HA and/or NA genes of different viruses [16]. It seemed to play the same role for the swPL01 virus. Even though some viruses appear to have reassorted with the pH1N1, the virus we characterized (swPL01) did not contain any gene segments derived from the pH1N1 (Figures 1, 2, and 3). Recently, H3N2v viruses were isolated in Korean swine [33], [34], and the M genes of swine H3N2 viruses from 2011–2012 indicated the pH1N1 M genes already reassorted with swine IAVs in Korea. To further investigate this relatedness, we analyzed the M genes of avian, human, and swine IAVs. In the inferred ML tree, we found that swine M genes were grouped into two big different clades (Figure S9). The first swine M clade was paralleled with that of humans and included the TRIG M lineage. The swPL01 M gene was placed in one of the evolution branches of this first swine M clade. However, the M genes of other Korean swine IAVs from 2011–2012 were found at the end of the pH1N1 M lineage, not directly from the second swine M clade below the avian Ms (Figure S9). These results could not reflect the overall M gene evolution of swine IAVs or explain the reassortment event of H3N2v in Korea. However, this might imply that the pH1N1 M segment would be more beneficial to the H3N2v gene constellation compared with that of avian and TRIG lineages (Figure S9). In fact, the pH1N1 virus adopted two gene segments, NA and M, from the EA lineage [35], [36], and both segments attributed to the enhanced viral release and transmission [37]. However, only the M gene appeared to reassort with other swine H3N2 viruses. This might be explained by the proposed pH1N1 M gene property for efficient transmission in a guinea pig model [38]. In this respect, the pandemic potential of the H3N2v virus [39] should be elucidated along with the contribution of pH1N1 NA segment for the generation of novel swine IAV variants [40]. Development of effective vaccines also needs for insightful preparedness against this ongoing epidemiological threat [41], [42].

For the HA and NA genes, the swPL01 virus belonged to the most recent evolution cluster in the swTx/98 phylogeny. So did the Korean isolates between 2011–2012 (Figures 2, S4, and S6). However, the phylogenetic analysis results inferred with avian, human, and swine H3N2 sequences indicated that the HA and NA genes of the swPL01 virus were categorized in different clades from those of other contemporary Korean isolates, respectively (Figures S4, S6, and S9). 35 and 29 amino acid mutations in the respective HA and NA segments also explained their phylogenetic distances from other Korean isolates (Tables S1 and S2). In addition, the swPL01 virus retained another potential NLG site in the HA1 region (Table S1). However, there were no avian H3 molecular signatures (Q226/G228) found in the HA of the swPL01 virus (V226/S228) (Table S1) even though some avian HAs were closely located together (Figure S9) [43], [44].

The swPL01 virus shared an overall 87.3–97.5% sequence similarity with the Korean swine H3N2 viruses. The highest sequence similarity of the swPL01 virus was found primarily with the North American TRIG H3N2 viruses circulating around 2005, except for the HA gene, which exhibited the highest similarity to that around 2011. Reassortment event(s) around 2011 might introduce the cluster IV HA, which was distinct from those of other contemporary Korean viruses, into the swPL01 virus. Considered all together, these results suggest that the swPL01 virus is one of the isolates evolved from the swTx/98 virus and that the diversity of swine IAVs circulating in Korea should be under close surveillance for pandemic preparedness.

## Supporting Information

Figure S1 **The phylogenetic relationships of the swPL01 PB2 gene with references.** The phylogenetic placement of the swPL01 PB2 gene was inferred by the ML method with PB2 reference sequences of swine H3N2 viruses. For the Maximum Parsimony (MP) tree, the selected nucleotide sequences were analyzed by the percentage of replicate (500 replicates), and the tree was inferred using the Subtree-Pruning-Regrafting algorithm with search level 1 where the initial trees were produced by the random sequence addition (10 replicates) [45]. Branch lengths calculated using the average pathway method were proportional for the units of the number of changes over the whole sequence. The Neighbor-Joining tree was also determined using the bootstrap method (500 replicates) [46]. Branch lengths were proportional for the same units as those of the evolutionary distance (the number of base substitutions per site) by the MCL method. Lineage definitions were indicated as: CS, classical swine; EA, Eurasian avian-like; and pH1N1, 2009 pandemic influenza A H1N1. The colors represent the following viruses: pink, A/swine/Texas/4199-2/1998 (swTx/98); green, Korean swine H3N2; red, A/swine/Korea/PL01/2012 (swPL01); and blue, 2009 pandemic influenza H1N1 viruses.
(TIF)

Figure S2 **The phylogenetic relationships of the swPL01 PB1 gene with references.** See Figure S1 legend.
(TIF)

Figure S3 **The phylogenetic relationships of the swPL01 PA gene with references.** See Figure S1 legend.
(TIF)

Figure S4 **The phylogenetic relationships of the swPL01 HA gene with references.** See Figure S1 legend. The HA phylogenetic groups were indicated as cluster I, II, III, and IV. The colors represent the following viruses: pink, A/swine/Texas/4199-2/1998 (swTx/98); green, Korean swine H3N2; and red, A/swine/Korea/PL01/2012 (swPL01).
(TIF)

Figure S5 **The phylogenetic relationships of the swPL01 NP gene with references.** See Figure S1 legend.
(TIF)

Figure S6 **The phylogenetic relationships of the swPL01 NA gene with references.** See Figure S1 legend. The NA phylogenetic groups were indicated as TRIG H3N2, human H1N2 (huH1N2), human H3N2 (huH3N2), and clade IV. The colors represent the following viruses: pink, A/swine/Texas/4199-2/1998 (swTx/98); green, Korean swine H3N2; and red, A/swine/Korea/PL01/2012 (swPL01).
(TIF)

Figure S7 **The phylogenetic relationships of the swPL01 M gene with references.** See Figure S1 legend.
(TIF)

Figure S8 **The phylogenetic relationships of the swPL01 NS gene with references.** See Figure S1 legend.
(TIF)

Figure S9 **The phylogenetic relationships of the swPL01 HA, NA, and M genes with avian, human, and swine references.** To determine the evolutionary placement of the swPL01 virus in animal and human H3N2 viruses, the phylogenetic relationships of swPL01 HA and NA genes were inferred by the ML method using avian (# of HAs, 37 Asian and 89 North American sequences; # of NAs, 62 Asian and 79 North American sequences), swine (# of HAs, 94 Asian and 413 North American sequences; # of NAs, 77 Asian and 426 North American sequences), and human (1,086 HA and 1,367 NA sequences of Asia) full-length sequences obtained from the NCBI database as of release date June 29, 2013. For the M gene analysis, a total of 3,707 sequences (# of avian Ms, 98 Asian and 797 North American sequences; # of swine Ms, 461 Asian and 958 North American sequences; and # of human Ms, 1,393 Asian sequences) from H1 and H3 subtype viruses were used in the ML method. Canine or feline HA, NA, and M gene sequences were also included together with those of avian sequences. The colors represent the following viruses: orange, avian; pale yellow, canine/feline; purple, human; forest green, swine; and blue, pH1N1. A/swine/Texas/4199-2/1998 (pink), Korean swine H3N2 isolates around 2011–2012 (green), and A/swine/Korea/PL01/2012 (red) were also indicated in the phylogenetic trees.
(TIF)

Table S1 **Comparison of the total HA amino acid sequences of swPL01 and Korean swine H3N2 (from 2011**–**2012) viruses.**
(TIF)

Table S2 **Comparison of the total NA amino acid sequences of swPL01 and Korean swine H3N2 (from 2011**–**2012) viruses.**
(TIF)

## References

[1] Webster RG, Bean WJ, Gorman OT, Chambers TM, Kawaoka Y (1992) Evolution and ecology of influenza A viruses. Microbiol Rev 56: 152–179. 1579108 10.1128/mr.56.1.152-179.1992 PMC372859

[2] Smith GJ, Vijaykrishna D, Bahl J, Lycett SJ, Worobey M, et al (2009) Origins and evolutionary genomics of the 2009 swine-origin H1N1 influenza A epidemic. Nature 459: 1122–1125. 19516283 10.1038/nature08182

[3] Kim JI, Lee I, Park S, Hwang MW, Bae JY, et al (2013) Genetic requirement for hemagglutinin glycosylation and its implications for influenza A H1N1 virus evolution. J Virol 87: 7539–7549. 23637398 10.1128/JVI.00373-13 PMC3700310

[4] Webster RG, Hulse DJ (2004) Microbial adaptation and change: avian influenza. Rev Sci Tech 23: 453–465. 15702713 10.20506/rst.23.2.1493

[5] Webby RJ, Webster RG (2001) Emergence of influenza A viruses. Philos Trans R Soc Lond B Biol Sci 356: 1817–1828. 11779380 10.1098/rstb.2001.0997 PMC1088557

[6] Ito T, Couceiro JN, Kelm S, Baum LG, Krauss S, et al (1998) Molecular basis for the generation in pigs of influenza A viruses with pandemic potential. J Virol 72: 7367–7373. 9696833 10.1128/jvi.72.9.7367-7373.1998 PMC109961

[7] Scholtissek C (1990) Pigs as the “mixing vessel” for the creation of new pandemic influenza A viruses. Med Principles Pract: 65–71.

[8] Reid AH, Fanning TG, Hultin JV, Taubenberger JK (1999) Origin and evolution of the 1918 “Spanish” influenza virus hemagglutinin gene. Proc Natl Acad Sci U S A 96: 1651–1656. 9990079 10.1073/pnas.96.4.1651 PMC15547

[9] Zhou NN, Senne DA, Landgraf JS, Swenson SL, Erickson G, et al (1999) Genetic reassortment of avian, swine, and human influenza A viruses in American pigs. J Virol 73: 8851–8856. 10482643 10.1128/jvi.73.10.8851-8856.1999 PMC112910

[10] Karasin AI, Schutten MM, Cooper LA, Smith CB, Subbarao K, et al (2000) Genetic characterization of H3N2 influenza viruses isolated from pigs in North America, 1977-1999: evidence for wholly human and reassortant virus genotypes. Virus Res 68: 71–85. 10930664 10.1016/s0168-1702(00)00154-4

[11] Vincent AL, Ma W, Lager KM, Janke BH, Richt JA (2008) Swine influenza viruses a North American perspective. Adv Virus Res 72: 127–154. 19081490 10.1016/S0065-3527(08)00403-X

[12] Brockwell-Staats C, Webster RG, Webby RJ (2009) Diversity of influenza viruses in swine and the emergence of a novel human pandemic influenza A (H1N1). Influenza Other Respi Viruses 3: 207–213. 10.1111/j.1750-2659.2009.00096.x PMC2746644 19768134

[13] Pappaioanou M, Gramer M (2010) Lessons from pandemic H1N1 2009 to improve prevention, detection, and response to influenza pandemics from a One Health perspective. ILAR J 51: 268–280. 21131728 10.1093/ilar.51.3.268 PMC7314042

[14] Trebbien R, Bragstad K, Larsen LE, Nielsen J, Botner A, et al (2013) Genetic and biological characterisation of an avian-like H1N2 swine influenza virus generated by reassortment of circulating avian-like H1N1 and H3N2 subtypes in Denmark. Virol J 10: 290. 24047399 10.1186/1743-422X-10-290 PMC3851529

[15] Centers for Disease C, Prevention (2011) Swine-origin influenza A (H3N2) virus infection in two children—Indiana and Pennsylvania, July-August 2011. MMWR Morb Mortal Wkly Rep 60: 1213–1215. 21900876

[16] Nelson MI, Vincent AL, Kitikoon P, Holmes EC, Gramer MR (2012) Evolution of novel reassortant A/H3N2 influenza viruses in North American swine and humans, 2009-2011. J Virol 86: 8872–8878. 22696653 10.1128/JVI.00259-12 PMC3421719

[17] Epperson S, Jhung M, Richards S, Quinlisk P, Ball L, et al (2013) Human Infections With Influenza A(H3N2) Variant Virus in the United States, 2011-2012. Clin Infect Dis 57 Suppl 1S4–S11. 23794729 10.1093/cid/cit272

[18] Biggerstaff M, Reed C, Epperson S, Jhung MA, Gambhir M, et al (2013) Estimates of the Number of Human Infections With Influenza A(H3N2) Variant Virus, United States, August 2011-April 2012. Clin Infect Dis 57 Suppl 1S12–15. 23794726 10.1093/cid/cit273 PMC4603749

[19] Vincent A, Awada L, Brown I, Chen H, Claes F, et al. (2013) Review of Influenza A Virus in Swine Worldwide: A Call for Increased Surveillance and Research. Zoonoses Public Health. 10.1111/zph.12049 23556412

[20] Vijaykrishna D, Smith GJ, Pybus OG, Zhu H, Bhatt S, et al (2011) Long-term evolution and transmission dynamics of swine influenza A virus. Nature 473: 519–522. 21614079 10.1038/nature10004

[21] Nelson MI, Lemey P, Tan Y, Vincent A, Lam TT, et al (2011) Spatial dynamics of human-origin H1 influenza A virus in North American swine. PLoS Pathog 7: e1002077. 21695237 10.1371/journal.ppat.1002077 PMC3111536

[22] Tamura K, Peterson D, Peterson N, Stecher G, Nei M, et al (2011) MEGA5: molecular evolutionary genetics analysis using maximum likelihood, evolutionary distance, and maximum parsimony methods. Mol Biol Evol 28: 2731–2739. 21546353 10.1093/molbev/msr121 PMC3203626

[23] Edgar RC (2004) MUSCLE: multiple sequence alignment with high accuracy and high throughput. Nucleic Acids Res 32: 1792–1797. 15034147 10.1093/nar/gkh340 PMC390337

[24] Felsenstein J (1985) Confidence limits on phylogenies: An approach using the bootstrap. Evolution 39: 783–791. 28561359 10.1111/j.1558-5646.1985.tb00420.x

[25] Pascua PN, Song MS, Lee JH, Choi HW, Han JH, et al (2008) Seroprevalence and genetic evolutions of swine influenza viruses under vaccination pressure in Korean swine herds. Virus Res 138: 43–49. 18789984 10.1016/j.virusres.2008.08.005

[26] Takemae N, Nguyen T, Ngo LT, Hiromoto Y, Uchida Y, et al (2013) Antigenic variation of H1N1, H1N2 and H3N2 swine influenza viruses in Japan and Vietnam. Arch Virol 158: 859–876. 23435952 10.1007/s00705-013-1616-8

[27] Richt JA, Lager KM, Janke BH, Woods RD, Webster RG, et al (2003) Pathogenic and antigenic properties of phylogenetically distinct reassortant H3N2 swine influenza viruses cocirculating in the United States. J Clin Microbiol 41: 3198–3205. 12843064 10.1128/JCM.41.7.3198-3205.2003 PMC165376

[28] Olsen CW, Karasin AI, Carman S, Li Y, Bastien N, et al (2006) Triple reassortant H3N2 influenza A viruses, Canada, 2005. Emerg Infect Dis 12: 1132–1135. 16836834 10.3201/eid1207.060268 PMC3291069

[29] Centers for Disease C, Prevention (2012) Update: Influenza A (H3N2)v transmission and guidelines - five states, 2011. MMWR Morb Mortal Wkly Rep 60: 1741–1744. 22217624

[30] Lee JH, Pascua PN, Song MS, Baek YH, Kim CJ, et al (2009) Isolation and genetic characterization of H5N2 influenza viruses from pigs in Korea. J Virol 83: 4205–4215. 19359528 10.1128/JVI.02403-08 PMC2668473

[31] Pascua PN, Song MS, Lee JH, Baek YH, Kwon HI, et al (2012) Virulence and transmissibility of H1N2 influenza virus in ferrets imply the continuing threat of triple-reassortant swine viruses. Proc Natl Acad Sci U S A 109: 15900–15905. 23019374 10.1073/pnas.1205576109 PMC3465388

[32] Choi YK, Pascua PN, Song MS (2013) Swine influenza viruses: an Asian perspective. Curr Top Microbiol Immunol 370: 147–172. 22266639 10.1007/82_2011_195

[33] Kim SH, Kim HJ, Jin YH, Yeoul JJ, Lee KK, et al. (2013) Isolation of influenza A(H3N2)v virus from pigs and characterization of its biological properties in pigs and mice. Arch Virol. 10.1007/s00705-012-1571-9 23674250

[34] Pascua PN, Lim GJ, Kwon HI, Park SJ, Kim EH, et al. (2013) Emergence of H3N2pM-like and novel reassortant H3N1 swine viruses possessing segments derived from the A (H1N1)pdm09 influenza virus, Korea. Influenza Other Respi Viruses. 10.1111/irv.12154 PMC4634262 24034626

[35] Neumann G, Noda T, Kawaoka Y (2009) Emergence and pandemic potential of swine-origin H1N1 influenza virus. Nature 459: 931–939. 19525932 10.1038/nature08157 PMC2873852

[36] Neumann G, Kawaoka Y (2011) The first influenza pandemic of the new millennium. Influenza Other Respi Viruses 5: 157–166. 10.1111/j.1750-2659.2011.00231.x PMC3073629 21477134

[37] Lakdawala SS, Lamirande EW, Suguitan AL Jr, Wang W, Santos CP, et al (2011) Eurasian-origin gene segments contribute to the transmissibility, aerosol release, and morphology of the 2009 pandemic H1N1 influenza virus. PLoS Pathog 7: e1002443. 22241979 10.1371/journal.ppat.1002443 PMC3248560

[38] Chou YY, Albrecht RA, Pica N, Lowen AC, Richt JA, et al (2011) The M segment of the 2009 new pandemic H1N1 influenza virus is critical for its high transmission efficiency in the guinea pig model. J Virol 85: 11235–11241. 21880744 10.1128/JVI.05794-11 PMC3194962

[39] Feng Z, Gomez J, Bowman AS, Ye J, Long LP, et al (2013) Antigenic Characterization of H3N2 Influenza A Viruses from Ohio Agricultural Fairs. J Virol 87: 7655–7667. 23637412 10.1128/JVI.00804-13 PMC3700273

[40] Kim JI, Lee I, Park S, Park MS (2012) Surface glycoproteins determine the feature of the 2009 pandemic H1N1 virus. BMB Rep 45: 653–658. 23187005 10.5483/BMBRep.2012.45.11.137 PMC4133798

[41] Loving CL, Lager KM, Vincent AL, Brockmeier SL, Gauger PC, et al. (2013) Efficacy of inactivated and live-attenuated influenza virus vaccines in pigs against infection and transmission of emerging H3N2 similar to the 2011–2012 H3N2v. J Virol. 10.1128/JVI.01038-13 PMC3754103 23824815

[42] Lee I, Kim JI, Park M-S (2013) Cell culture-based Influenza Vaccines as Alternatives to Egg-based Vaccines. Journal of Bacteriology and Virology 43: 9–17.

[43] Rogers GN, Paulson JC, Daniels RS, Skehel JJ, Wilson IA, et al (1983) Single amino acid substitutions in influenza haemagglutinin change receptor binding specificity. Nature 304: 76–78. 6191220 10.1038/304076a0

[44] Connor RJ, Kawaoka Y, Webster RG, Paulson JC (1994) Receptor specificity in human, avian, and equine H2 and H3 influenza virus isolates. Virology 205: 17–23. 7975212 10.1006/viro.1994.1615

[45] Nei M, Kumar S (2000) Molecular evolution and phylogenetics. New York: Oxford University Press.

[46] Saitou N, Nei M (1987) The neighbor-joining method: A new method for reconstructing phylogenetic trees. Molecular Biology and Evolution 4: 406–425. 3447015 10.1093/oxfordjournals.molbev.a040454

