# Supplementary figures and images for: Phylogenetic Analysis of a Swine Influenza A(H3N2) Virus Isolated in Korea in 2012
Source: PLoS One. 2014 Feb 11;9(2):e88782. doi: 10.1371/journal.pone.0088782 (PMC3921248; doi:10.1371/journal.pone.0088782)

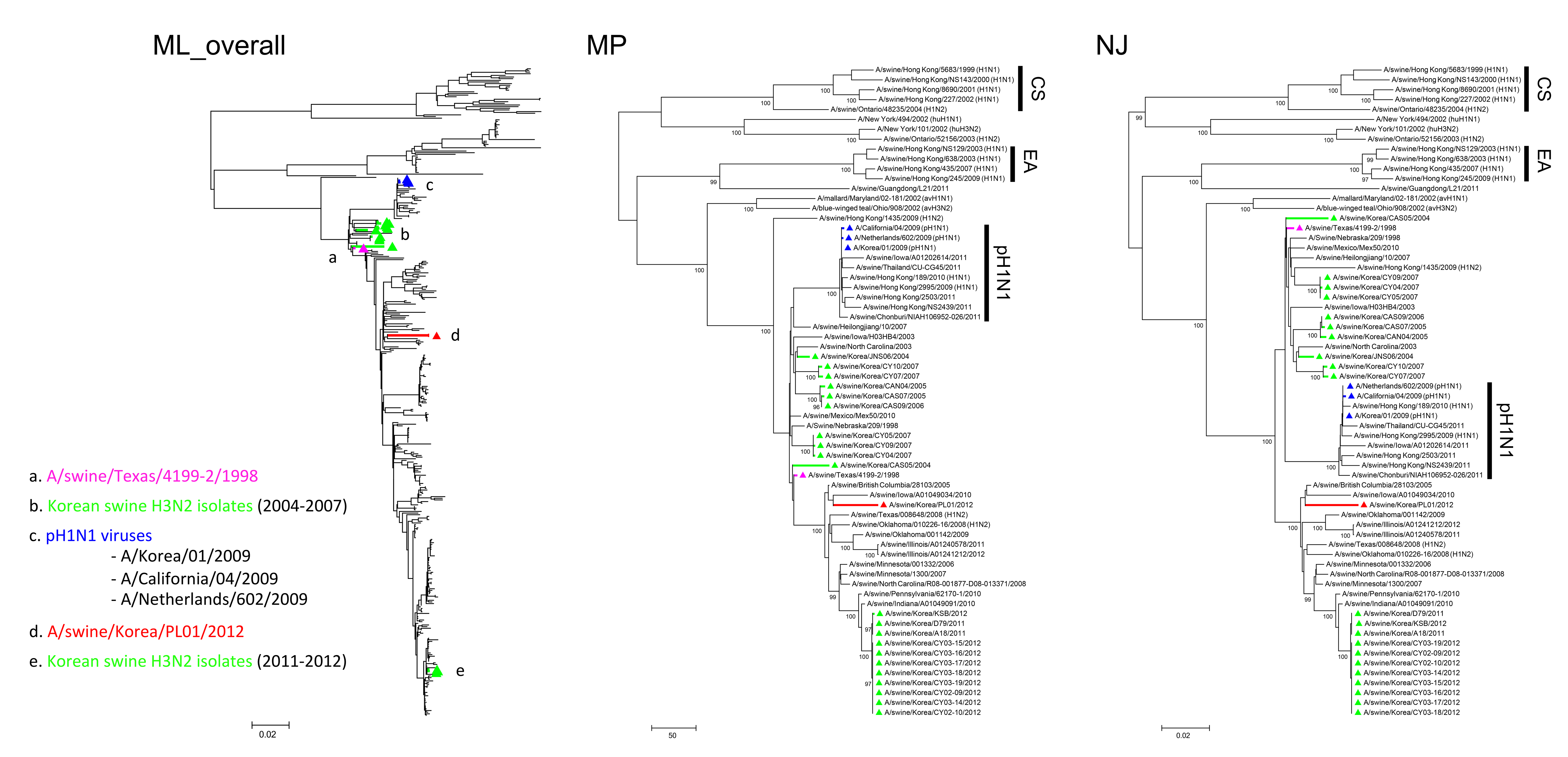

Supplement: Figure S1 — The phylogenetic relationships of the swPL01 PB2 gene with references. The phylogenetic placement of the swPL01 PB2 gene was inferred by the ML method with PB2 reference sequences of swine H3N2 viruses. For the Maximum Parsimony (MP) tree, the selected nucleotide sequences were analyzed by the percentage of replicate (500 replicates), and the tree was inferred using the Subtree-Pruning-Regrafting algorithm with search level 1 where the initial trees were produced by the random sequence addition (10 replicates) [45]. Branch lengths calculated using the average pathway method were proportional for the units of the number of changes over the whole sequence. The Neighbor-Joining tree was also determined using the bootstrap method (500 replicates) [46]. Branch lengths were proportional for the same units as those of the evolutionary distance (the number of base substitutions per site) by the MCL method. Lineage definitions were indicated as: CS, classical swine; EA, Eurasian avian-like; and pH1N1, 2009 pandemic influenza A H1N1. The colors represent the following viruses: pink, A/swine/Texas/4199-2/1998 (swTx/98); green, Korean swine H3N2; red, A/swine/Korea/PL01/2012 (swPL01); and blue, 2009 pandemic influenza H1N1 viruses. (TIF) [file pone.0088782.s001.tif]

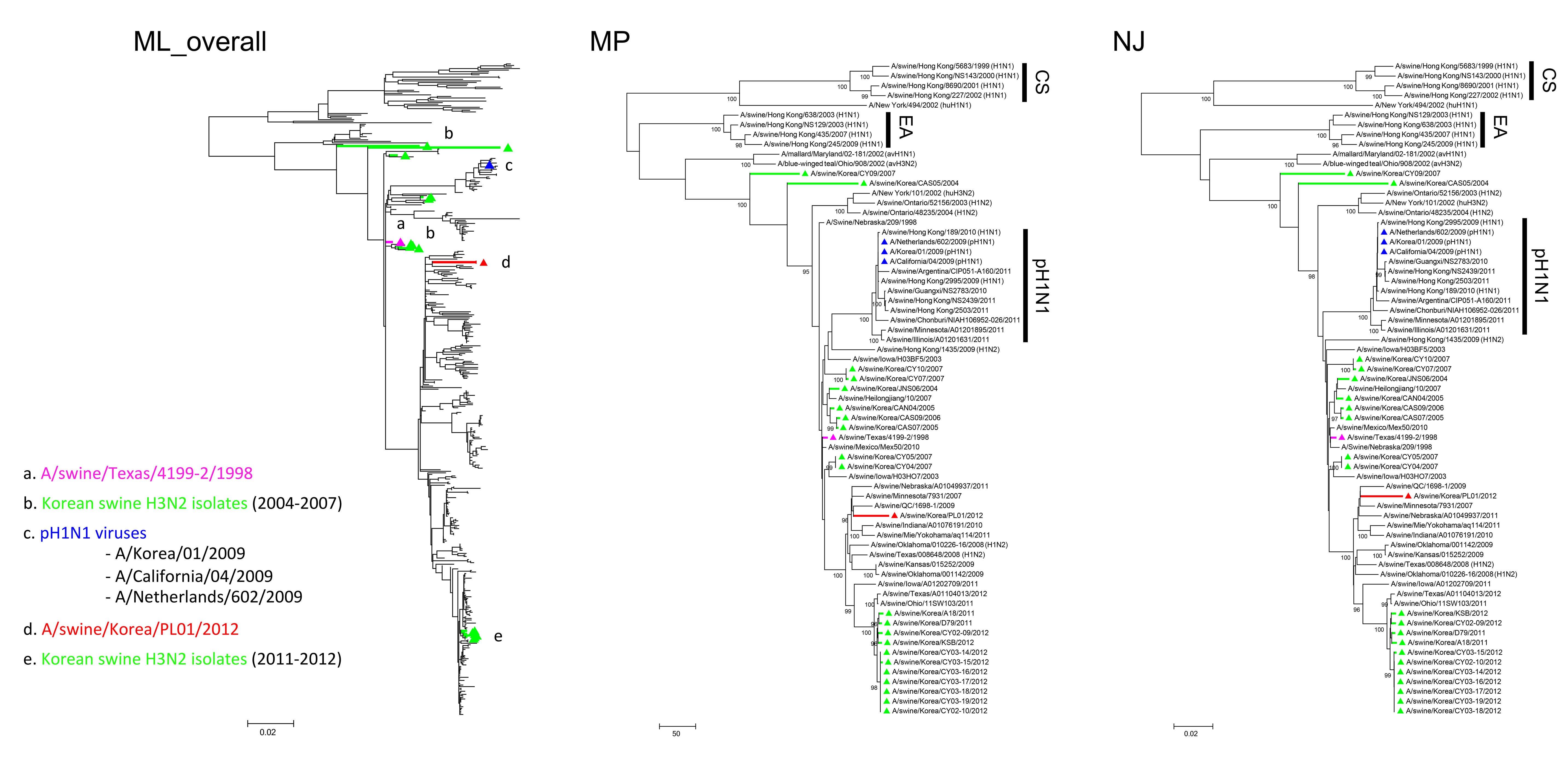

Supplement: Figure S2 — The phylogenetic relationships of the swPL01 PB1 gene with references. See Figure S1 legend. (TIF) [file pone.0088782.s002.tif]

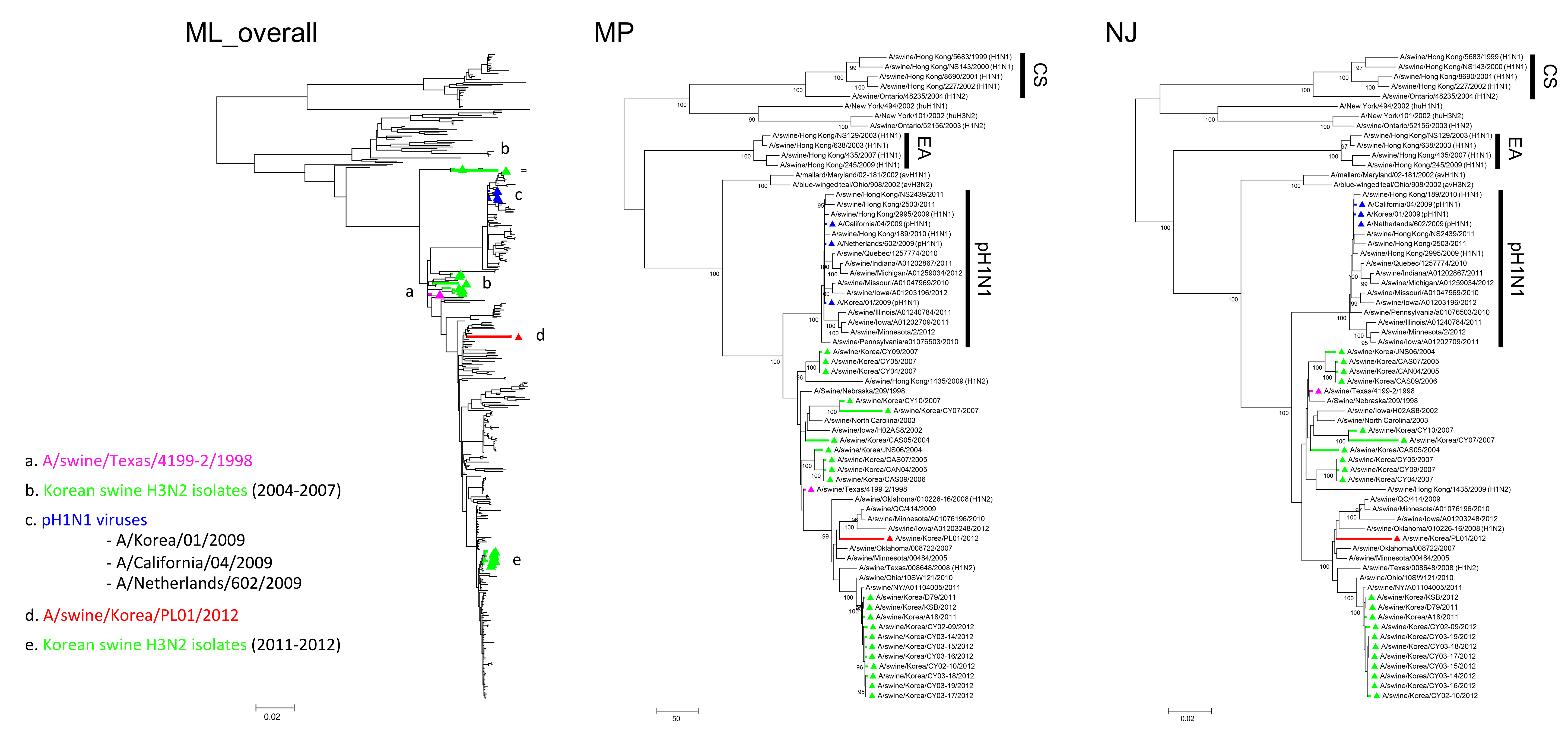

Supplement: Figure S3 — The phylogenetic relationships of the swPL01 PA gene with references. See Figure S1 legend. (TIF) [file pone.0088782.s003.tif]

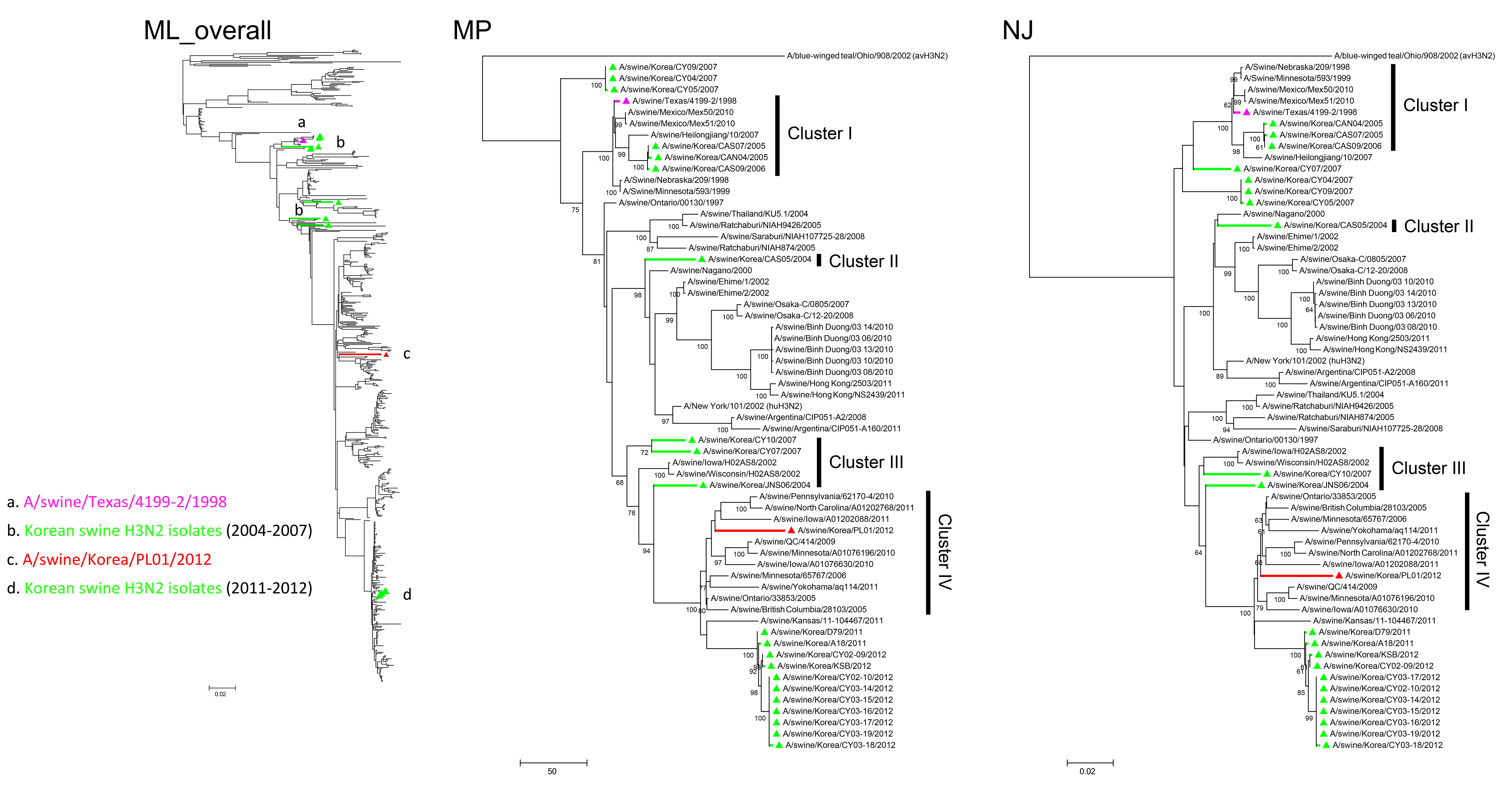

Supplement: Figure S4 — The phylogenetic relationships of the swPL01 HA gene with references. See Figure S1 legend. The HA phylogenetic groups were indicated as cluster I, II, III, and IV. The colors represent the following viruses: pink, A/swine/Texas/4199-2/1998 (swTx/98); green, Korean swine H3N2; and red, A/swine/Korea/PL01/2012 (swPL01). (TIF) [file pone.0088782.s004.tif]

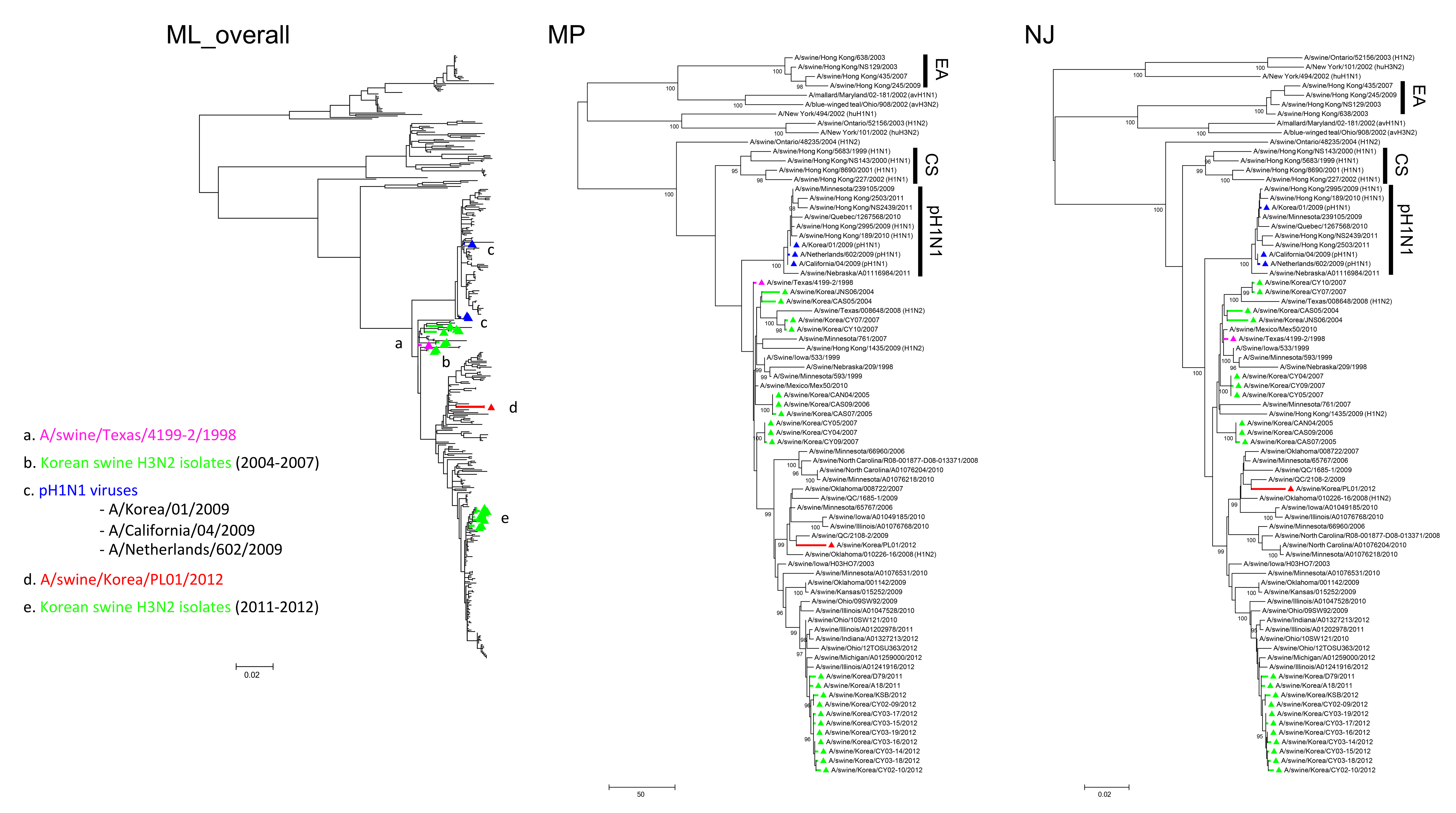

Supplement: Figure S5 — The phylogenetic relationships of the swPL01 NP gene with references. See Figure S1 legend. (TIF) [file pone.0088782.s005.tif]

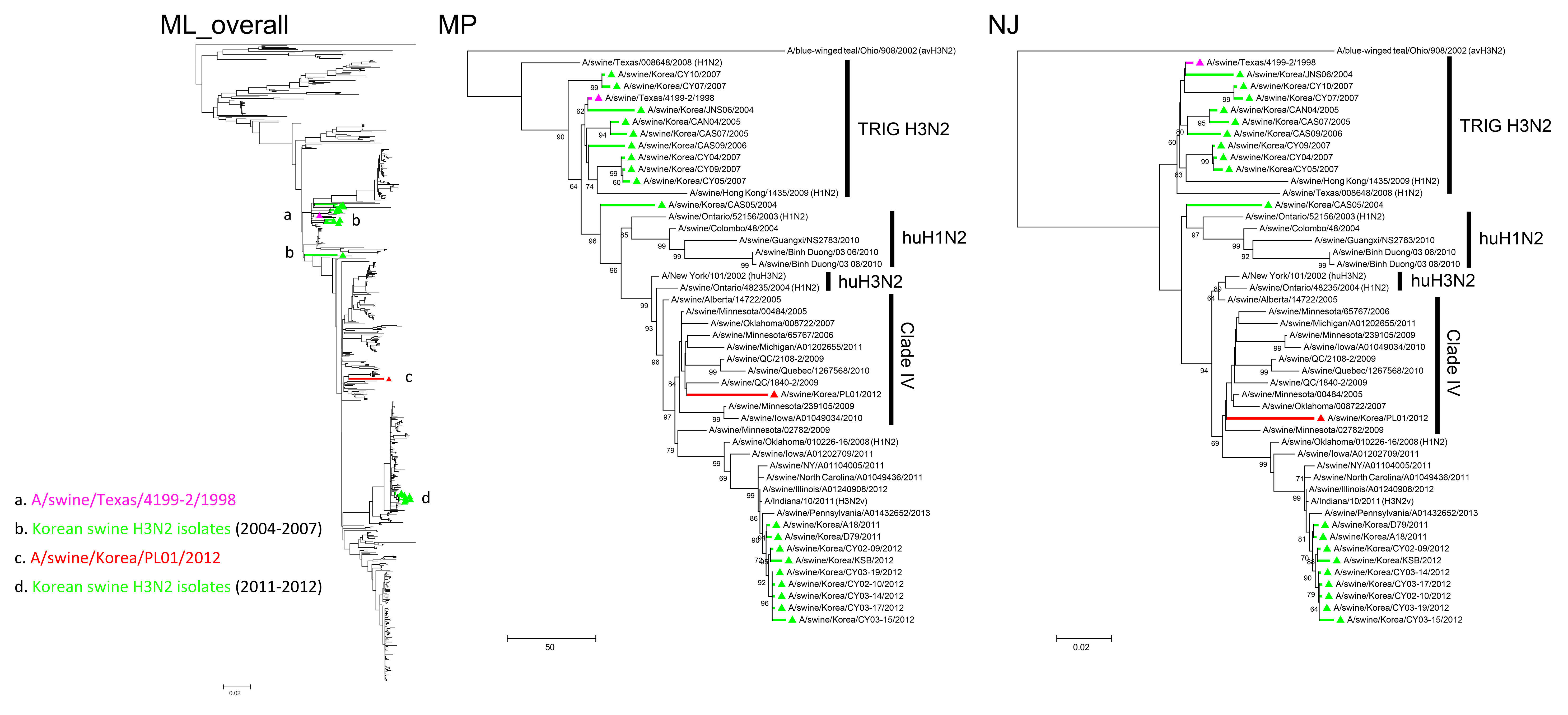

Supplement: Figure S6 — The phylogenetic relationships of the swPL01 NA gene with references. See Figure S1 legend. The NA phylogenetic groups were indicated as TRIG H3N2, human H1N2 (huH1N2), human H3N2 (huH3N2), and clade IV. The colors represent the following viruses: pink, A/swine/Texas/4199-2/1998 (swTx/98); green, Korean swine H3N2; and red, A/swine/Korea/PL01/2012 (swPL01). (TIF) [file pone.0088782.s006.tif]

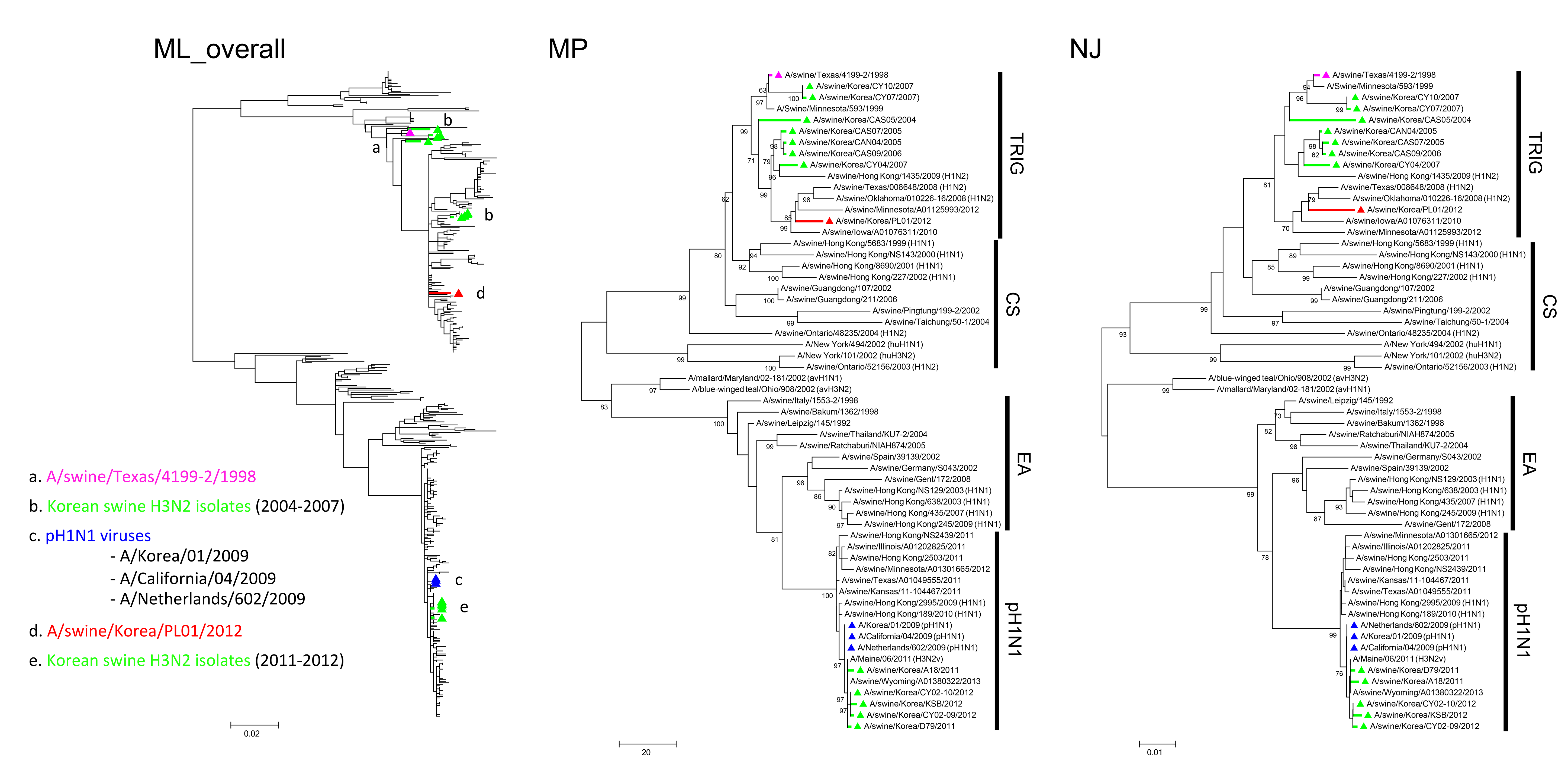

Supplement: Figure S7 — The phylogenetic relationships of the swPL01 M gene with references. See Figure S1 legend. (TIF) [file pone.0088782.s007.tif]

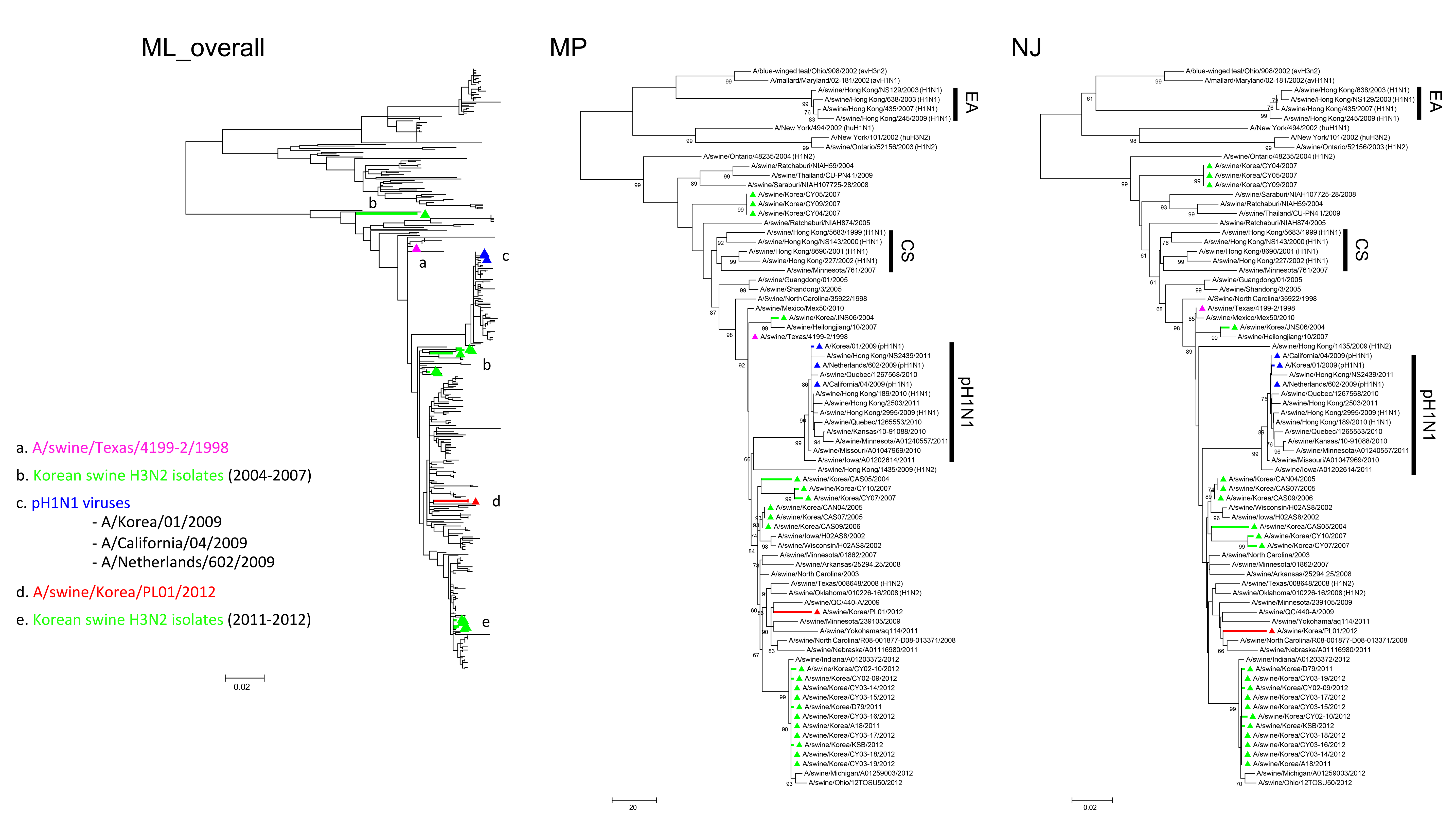

Supplement: Figure S8 — The phylogenetic relationships of the swPL01 NS gene with references. See Figure S1 legend. (TIF) [file pone.0088782.s008.tif]

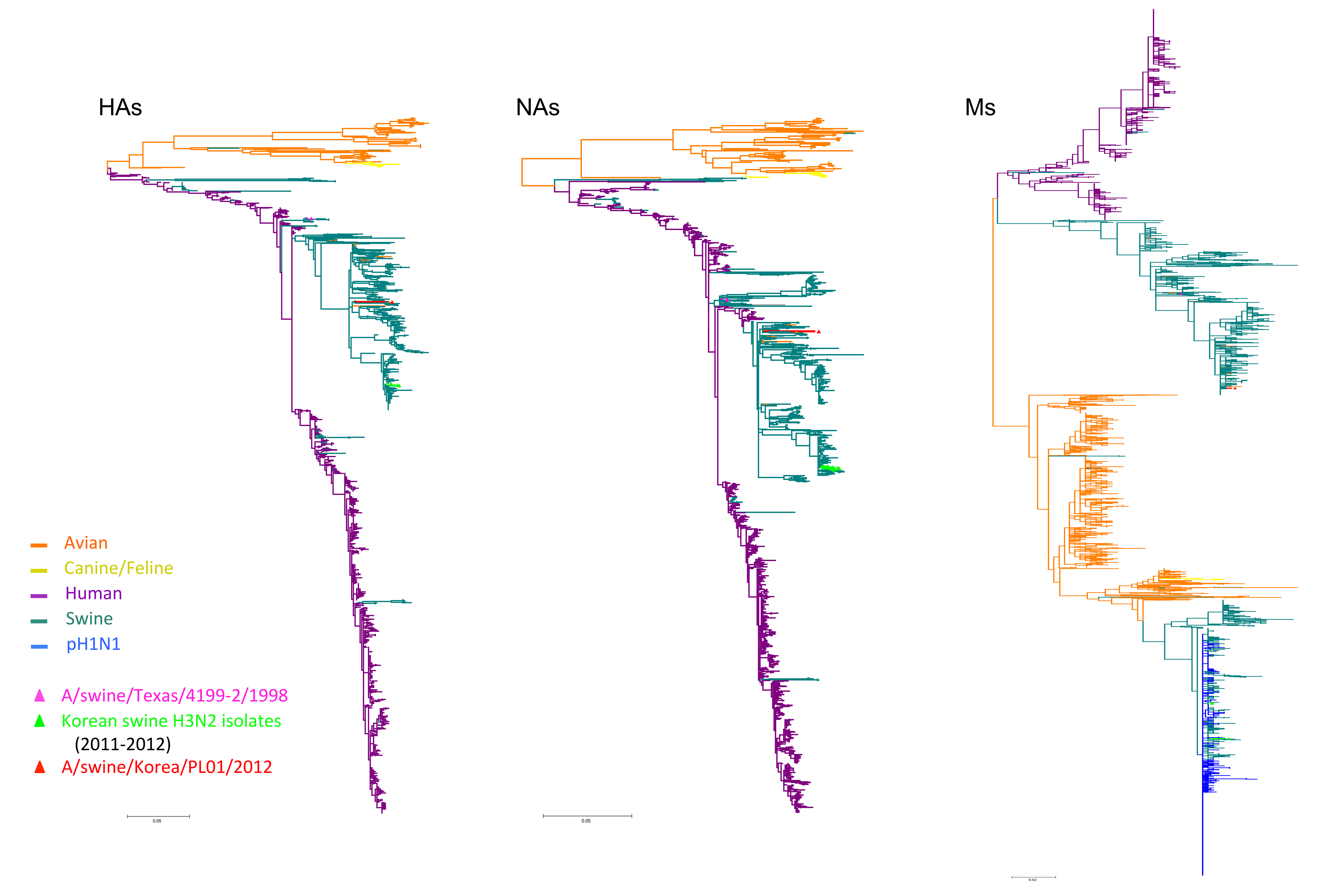

Supplement: Figure S9 — The phylogenetic relationships of the swPL01 HA, NA, and M genes with avian, human, and swine references. To determine the evolutionary placement of the swPL01 virus in animal and human H3N2 viruses, the phylogenetic relationships of swPL01 HA and NA genes were inferred by the ML method using avian (# of HAs, 37 Asian and 89 North American sequences; # of NAs, 62 Asian and 79 North American sequences), swine (# of HAs, 94 Asian and 413 North American sequences; # of NAs, 77 Asian and 426 North American sequences), and human (1,086 HA and 1,367 NA sequences of Asia) full-length sequences obtained from the NCBI database as of release date June 29, 2013. For the M gene analysis, a total of 3,707 sequences (# of avian Ms, 98 Asian and 797 North American sequences; # of swine Ms, 461 Asian and 958 North American sequences; and # of human Ms, 1,393 Asian sequences) from H1 and H3 subtype viruses were used in the ML method. Canine or feline HA, NA, and M gene sequences were also included together with those of avian sequences. The colors represent the following viruses: orange, avian; pale yellow, canine/feline; purple, human; forest green, swine; and blue, pH1N1. A/swine/Texas/4199-2/1998 (pink), Korean swine H3N2 isolates around 2011–2012 (green), and A/swine/Korea/PL01/2012 (red) were also indicated in the phylogenetic trees. (TIF) [file pone.0088782.s009.tif]

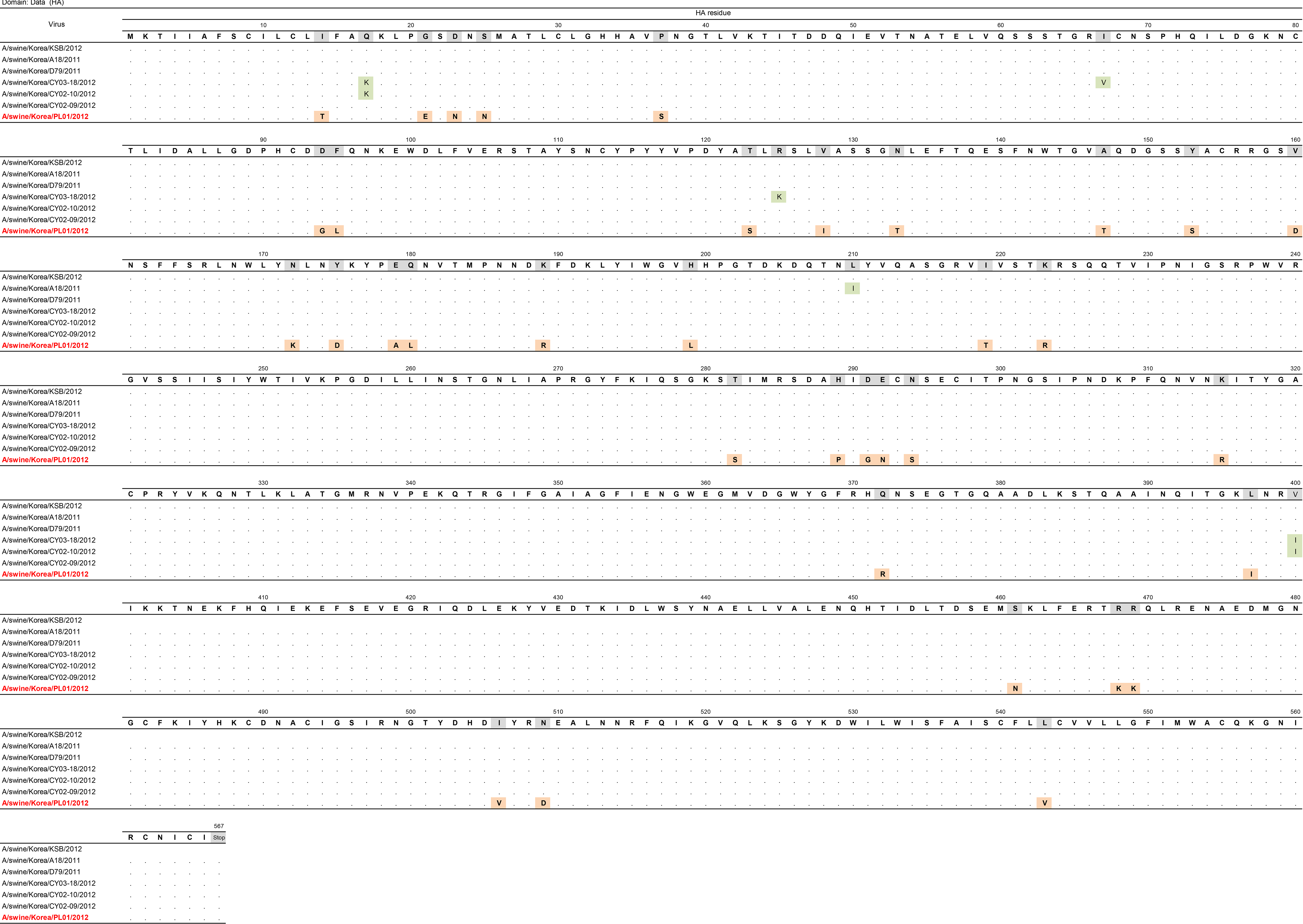

Supplement: Table S1 — Comparison of the total HA amino acid sequences of swPL01 and Korean swine H3N2 (from 2011–2012) viruses. (TIF) [file pone.0088782.s010.tif]

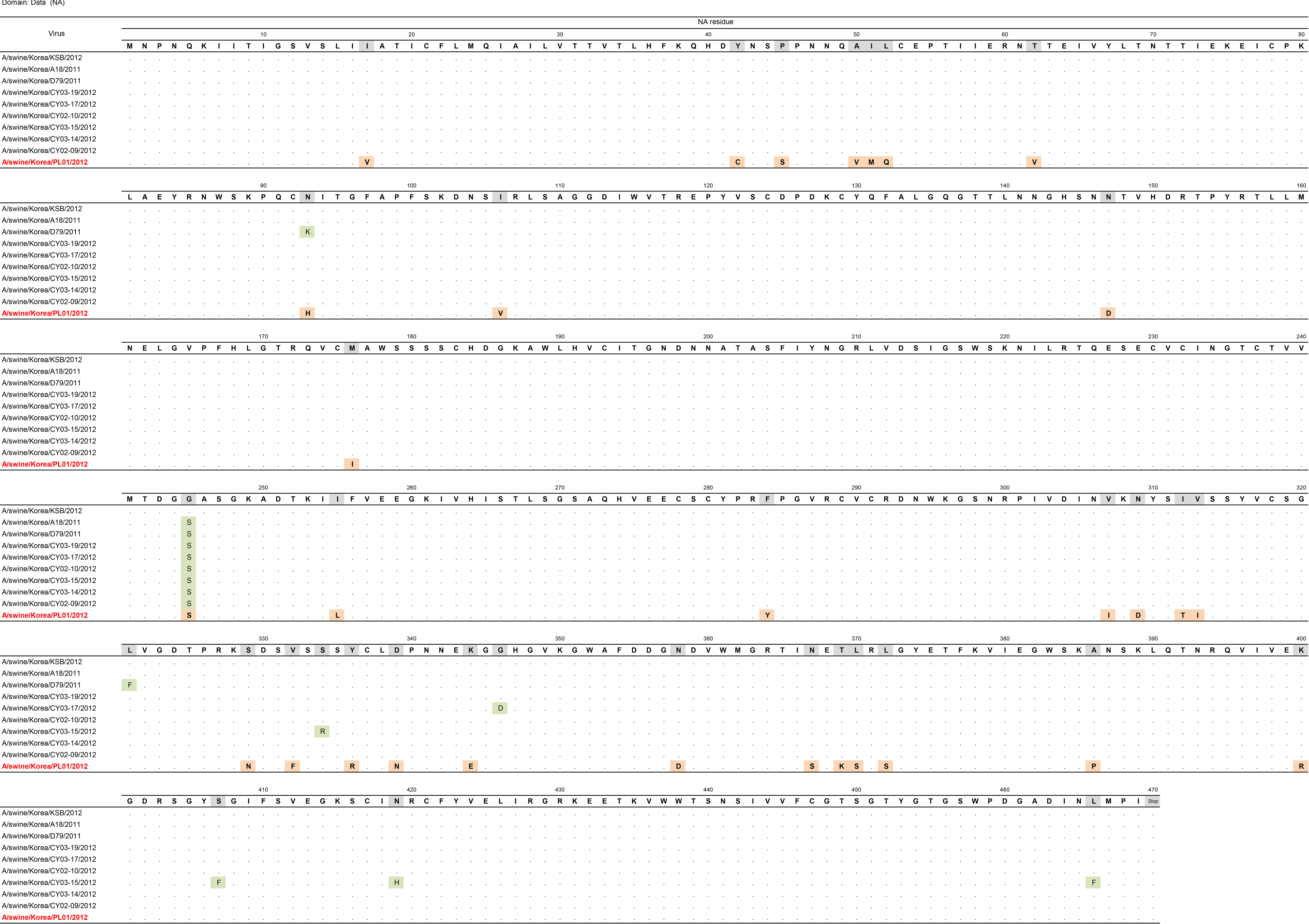

Supplement: Table S2 — Comparison of the total NA amino acid sequences of swPL01 and Korean swine H3N2 (from 2011–2012) viruses. (TIF) [file pone.0088782.s011.tif]
